# Is the mandibular buccal shelf anatomy related to craniofacial morphology? A cross-sectional CBCT study

**DOI:** 10.4317/medoral.26897

**Published:** 2024-12-24

**Authors:** María Dolores Campoy, Salvador Chiquillo-Enguix, Verónica García-Sanz, Juan Carlos Pérez-Varela, Sara Camañes-Gonzalvo, Vanessa Paredes-Gallardo

**Affiliations:** 1 Department of Stomatology, University of Valencia, Spain; 2 Private Practice. Clínica Pérez Varela Maex, Santiago de Compostela, Spain

## Abstract

**Background:**

The placement of Temporary Anchorage Devices (TADs) in the mandibular buccal shelf area is a common option for distalizing the lower arch. Therefore, the study of bone thickness and depth in this area is mandatory before planning TAD insertion. The aim of this study was to quantify the width and depth of the mandibular buccal shelf structure and examine its associations with sex, age, skeletal class and vertical pattern.

**Material and Methods:**

A cross-sectional study was carried out on cone beam computed tomographies obtained from 91 patients. The bone thickness was evaluated in the mandibular buccal shelf area 5 and 8 mm apical to the cement-enamel junction (CEJ), and the bone depth was measured 4 mm buccal to the CEJ at the level of the distal root of the mandibular first molar and the mesial root of the mandibular second molar using the InVivoDental 6.0 software.

**Results:**

The depth and thickness of the bone increased in distal areas, and the thickness was greater at 8 mm. No differences were found between sex or skeletal class. Bone thickness decreased with age, and it was significantly lower in hyperdivergent patients.

**Conclusions:**

The thickness of the bone was higher in distal and deeper areas, and the depth was greater in distal areas. The hyperdivergent facial pattern and age were negatively associated with bone thickness.

** Key words:**Temporary anchorage devices (TADs), mandibular external oblique line, cone beam computed tomography (CBCT), buccal shelf.

## Introduction

Anchor management is crucial in orthodontic treatment planning. Temporary anchorage devices (TADs) have simplified treatment biomechanics and enabled complex dental movements (1-3). Distalizing the mandibular arch in Class III malocclusion cases is challenging due to posterior anatomical limitations (4). However, this approach can achieve significant sagittal changes and improve the lower facial profile without premolar extraction, offering a viable solution for such patients (5-9).

Since placing TADs in the buccal shelf is a common approach for distalization treatment (10), many studies have explored various methods to measure bone thickness and depth in this area. These studies aim to identify the ideal bone zone for safe and reliable TAD placement (11-23).

While the results of the studies have indicated that bone thickness increases distally from the first to the second molar (13,17,22), there is less consensus on depth measurements since some authors find that they increase (13,17,18), while others find the opposite results (22).

Some studies found no significant gender differences (18,20), while others reported variations favoring males (22) or females (21). There is also no consensus on age; generally, bone thickness and depth decrease with age (18,21). However, some research suggests that growing patients have greater bone width, while nongrowing patients have greater bone height (22).

Studies on skeletal class yielded varying results. Golshah *et al*. found greater bone thickness in Class II patients (15), while others found increased thickness and height in Class III patients (18,20). As for the vertical pattern, some studies found no correlation with bone thickness and depth (18), while others reported that hypodivergent patients have greater thickness (22) and depth (19,23). Additionally, one study noted that hyperdivergent patients have greater depth than hypodivergent patients (22).

The variability in study results and the limited research on how skeletal class and vertical pattern affect bone anatomy in the mandibular buccal shelf highlight the need for further investigation. Therefore, this study aims to measure and compare the width and depth of the mandibular buccal shelf bone near the first and second molars and examine correlations with age, gender, skeletal class, and vertical pattern.

## Material and Methods

- Overview

The present study was an observational, cross-sectional, and descriptive study. The study was approved by the Ethics Committee for Human Research at the University of Valencia (No. 1867515). The study followed the guidelines established by the Declaration of Helsinki for research involving human subjects, as well as the STROBE guidelines for observational studies.

- Participants

A total of 110 cone beam computed tomography (CBCT) scans from patients treated in the Orthodontic Master's program at the University of Valencia and at a private dental clinic were collected between March 2022 and December 2022. These CBCT scans were part of the patient’s initial records and had been taken for reasons unrelated to the current study. The following inclusion and exclusion criteria were applied to select the final sample.

The inclusion criteria were as follows: (1) full skull CBCT scan; and (2) presence of both first and second mandibular molars on both sides. The exclusion criteria were as follows: (1) low-quality CBCT scans; (2) impaction of first and/or second mandibular molars; (3) presence of implants or prosthetics in the position of the first and/or second mandibular molars; (4) periodontal disease or severe bone-affecting diseases; and (5) presence of facial asymmetries or evident craniofacial syndromes.

- CBCT measurements

The CBCT scans were taken in the natural head position using the Planmeca Promax 3D machine (Planmeca, Helsinki, Finland) (field of view 20x19 cm) with a voxel size of 0.4 mm. Invivo Dental 6 software (Anatomage, San Jose, CA, USA) was used for all linear measurements as well as for obtaining lateral cephalograms. Dolphin Imaging 11.95 Premium software was used for cephalometric analysis to determine skeletal class and vertical pattern.

The Ricketts XY axis angle was used to classify the vertical pattern (angle formed by the basicranial plane-Ba-Na and the line Pt-Gn). The norm was defined as 90° ± 3° (normodivergent pattern), > 93° = hypodivergent; < 87° = hyperdivergent pattern. Skeletal class was classified using the ANB angle (angle formed by Lines N-A and N-B) with a norm of 2° ± 2° (Skeletal Class I); > 4° = Skeletal Class II; < 0° = Skeletal Class III.

**Figure 1:**
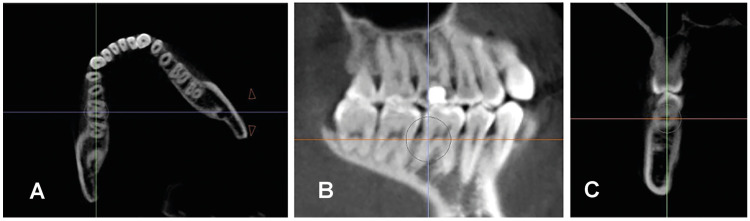
(A) Axial view, (B) Sagittal view, depicting the orientation for measurements on the distal root of the first mandibular molar. (C) Coronal view showing the distal root of the first mandibular molar where the measurements were taken. Images were obtained using Invivo 6 software.

The following orientation and selection protocol for the measurement plane was established:

1. Axial view: The plane was oriented so that the sagittal axis line passed through the midpoints of the mesial root of the first mandibular molar and the distal root of the second mandibular molar (Fig. 1).

2. Sagittal view: The image was oriented with the horizontal axis passing through the furcation point of the first and second mandibular molars, closest to the pulp chamber. The vertical axis was aligned with the distal root of the first mandibular molar or the mesial root of the second mandibular molar (Fig. 1).

3. Coronal view (Fig. 1): After orienting the image and selecting the measurement slice, the bone thickness and depth at the distal root of the first molar and mesial root of the second molar were calculated for both sides using the software's measurement tool.

**Figure 2:**
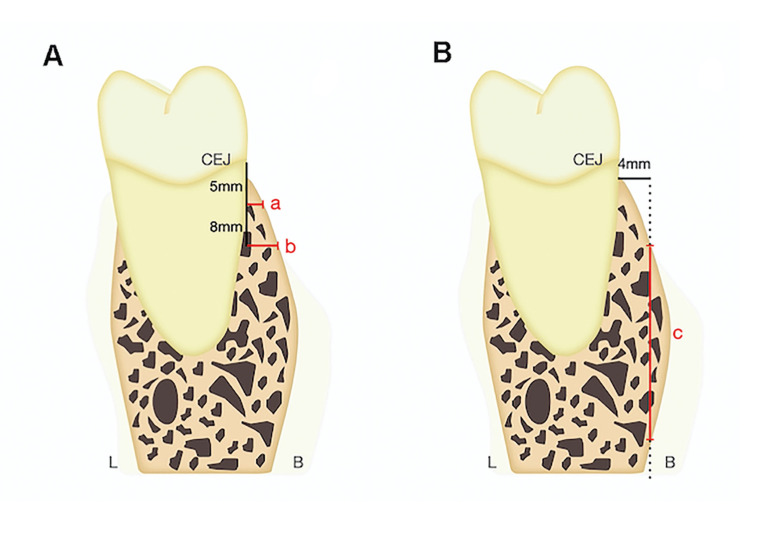
(A) Bone thickness measurement illustration at 5 mm (a) and 8 mm (b) from the CEJ. (B) Bone depth measurement illustration at 4 mm (c) buccally from the CEJ. Green line: vertical reference line.

Bone thickness measurements were taken by drawing a line through the cement-enamel junction (CEJ) parallel to the vertical reference line (green in Fig. 1). A perpendicular line was drawn 5 mm apical to the CEJ to the buccal point of the alveolar bone, and the distance was measured (measurement a in Fig. 2). The same method was used 8 mm apical to the CEJ (measurement b in Fig. 2).

For bone depth, a line was projected from the CEJ perpendicular to the vertical reference line and extended 4 mm buccally. The distance between the coronal and apical points contacting the cortical bone along this line was measured (measurement c in Fig. 2).

Measurements that could not be anatomically performed were deemed not measurable and excluded from the overall analysis (Supplement 1). These excluded data points were analyzed separately to check for relationships with the study variables.

- Statistical analysis

A minimum sample size of 90 patients was needed to detect significant thickness differences among the 3 facial types with a medium to large effect size (f=0.33) and 80% power. This size effect is equivalent to mean values of 2.5, 3, and 3.5 mm (SD=1.5), based on the study of Gandhi *et al*. (22). The analysis was conducted using one-way ANOVA with 95% confidence intervals.

The Kolmogorov-Smirnov test was used to assess normal distribution. An independent samples t-test compared means between two groups (sex), while one-way ANOVA compared means across multiple groups (patterns, age), with Bonferroni adjustments for multiple comparisons. The Spearman correlation coefficient evaluated the linear association between dimensions and age. The significance level was set at 5% (α=0.05). The independent samples t-test had a power of 86.9% for a medium-large effect size (d=0.65), and the paired samples t-test had 99.7% power for a medium effect size (d=0.5).

Two examiners (SCE, MCF) performed the measurements under standardized ambient conditions. To estimate the intraclass coefficient (ICC), measurements of 50% of the cuts were repeated one week later under the same conditions by both evaluators to determine intra- and interexaminer error.

## Results

After applying the inclusion and exclusion criteria, 19 CBCT scans were excluded (17.27%), resulting in a sample of 91 CBCT scans from 42 males (46.2%) and 49 females (53.8%), with a mean age of 28.6 ± 14.0 years. No evidence of sexual dimorphism was found. The distribution of the sample according to skeletal class and vertical pattern was homogeneous. Of these 91 CBCT scans, a total of 364 slices were evaluated, and 43 (23.62%) slices presented at least one nonmeasurable value.

The analysis of reproducibility showed high results, with ICC values exceeding 0.90 for all measurements, both for inter-examiner and intra-examiner errors.

- Thickness and depth

Bone thickness and depth showed a significant increase from distal to the first molar to mesial to the second molar. Significantly higher values were also found in the thickness measured at 8 mm than in the measurements at 5 mm (Table 1 and Supplement 2).

- Age and gender

No evidence of sexual dimorphism was found. There was an inverse correlation between the thickness variables and age, as thickness decreased with age (Supplement 3).

- Skeletal class

There was no generalized effect of skeletal class beyond a specific difference for T8-6Dr and T5-7Ml. In both cases, individuals with Class III exhibit thinner thickness (Supplement 4).

- Vertical Pattern

Significant differences were found in several measurements (Supplement 5). Essentially, the analysis highlighted the thickness differences between hyperdivergent individuals and the other two groups, particularly in comparison to normodivergent individuals (Table 2, Supplement 6 and Supplement 7).

- Measurement feasibility

Some measurements could not be performed due to anatomic issues. The depth variable distal to the first molar was the most strongly affected parameter (Supplement 8). The hyperdivergent pattern was significantly more affected than the normal and hypodivergent patte rns (Supplement 9).

## Discussion

Given the variability and lack of consensus on how skeletal class and vertical pattern relate to the buccal shelf area, this study aimed to investigate these factors in a homogeneous sample. This modified protocol was based on methods by Huang *et al*., Escobar-Correa *et al*., and Aleluia *et al*. (14,18,20). CBCT was used for its precision and reliability (24). The Ricketts XY axis angle was employed to classify the vertical pattern, avoiding the mismatches that can occur with the mandibular plane angle used in other studies (22).

Most studies used the CEJ as a reference point due to its high reproducibility (12-15,17-19,21,22), while some used the alveolar crest (11,16,20). The alveolar crest was excluded in this study due to its variability and susceptibility to bone loss. Bone thickness was measured 5 mm and 8 mm apical to the CEJ, despite variations in the literature (13,14,16-19). These distances were chosen because bone is often insufficient under 5 mm, and a miniscrew deeper than 8 mm would require a long transmucosal neck design.

Bone depth was measured 4 mm buccally from the CEJ, as this point is most standardized (17,18,23). Measurements further buccally may fall outside the bone, and distances under 4 mm are typically avoided for TAD placement to reduce the risk of root perforation (17,18).

Consistent with previous studies (1,2,14-17,21), bone thickness was lower at the distal root of the first molar compared to the mesial root of the second molar, and thickness was greater at 8 mm than at 5 mm. Given that TADs are usually 2 mm in diameter (16), a minimum of 4 mm of buccal bone is required for safety, although some suggest higher values without accounting for the tapering of both the root and TAD apically (17).

This study, consistent with previous research (17), found sufficient bone thickness for a 2 mm TAD only at the mesial root area of the second molar at 8 mm depth. Thus, this location is most advisable, provided the TAD design includes a suiTable transmucosal neck for this depth.

Consistent with previous research (17,18), greater bone depth was observed distally. Alveolar bone height measurements, both distal to the first molar (15.1 ± 4.83 right - 16.7 ± 5.26 left) and mesial to the second molar (17.4 ± 4.11 right - 18.9 ± 4.22 left), align with common TAD lengths. However, the distal first molar area had the highest percentage of nonmeasurable values. Escobar-Correa *et al*. (18) reported slightly lower values, particularly distal to the first molar, possibly due to data considered null in our study.

No significant sex-based differences in bone thickness or depth were found, in line with other studies (18,20). An inverse correlation between bone thickness and age was observed, consistent with previous research (18,21,22). Only two specific measurements showed lower thickness in Class III patients, unlike other studies that reported more generalized differences (15,18,20).

Bone thickness values were associated with the vertical pattern. Consistent with previous studies (20,22), hyperdivergent patients had significantly lower measurements compared to hypodivergent and normodivergent patients. However, this study found no significant differences between normodivergent and hypodivergent patients, contrary to other reports (20,22). Notably, Matias *et al*. observed higher measurements in hyperdivergent individuals at the mesial and distal areas of the second molars, though their sample size was small (19).

The study found no differences in bone depth among vertical patterns, aligning with some research (18), while other studies (22) reported greater depth in hyperdivergent patients. Notably, bone depth in the distal area of the first molars was not measurable in 53.3% (right) and 33.3% (left) of hyperdivergent patients, indicating that thickness and depth values in these cases can be more variable. Thus, individual analysis is crucial for hyperdivergent patients.

The study had limitations, including 43 slices with at least one nonmeasurable value (23.62%), and lacked comparisons with other studies due to differing methodologies. Additionally, it did not assess bone density or the cortical and trabecular thickness. Future research should include these factors for a more comprehensive analysis.

## Conclusions

1. Greater thickness values were found at the mesial area of the second molar at a distance of 8 mm from the CEJ.

2. No statistically significant differences were found between gender.

3. Older patients exhibited lower bone thickness, unrelated to bone depth.

4. The vertical pattern had the most significant impact on bone dimensions, with lower bone thickness values in hyperdivergent patients, while no generalized effect of antero-posterior skeletal class was found.

## Figures and Tables

**Table 1:** Dimensions of thickness and depth by tooth and distance to the cement-enamel junction (CEJ).

Root	Thickness (mm)		Depth (mm)
	5 mm (Mean ± SD)	8 mm (Mean ± SD)	4 mm (Mean ± SD)
6Dr	1.96 ± 0.99 (1.75-2.18)	2.92 ± 1.47 (2.61-3.23)	15.1 ± 4.83 (13.9-16.3)
7Mr	2.71 ± 1.61 (2.37-3.05)	4.38 ± 1.97 (3.97-4.79)	17.4 ± 4.11 (16.4-18.3)
6Dl	2.18 ± 1.14 (1.93-2.42)	3.38 ± 1.75 (3.01-3.75)	16.7 ± 5.26 (15.5-17.9)
7Ml	3.26 ± 1.98 (2.84-3.69)	5.09 ± 2.14 (4.64-5.54)	18.9 ± 4.22 (18.0-19.8)

Abbreviations. 6Dr: distal root of the first molar right; 7Mr: mesial root of the second molar right; 6Dl: distal root of the first molar left; 7Ml: mesial root of the second molar left; SD: standard deviation.

**Table 2:** Dimensions of thickness by tooth and vertical pattern.

Root	Thickness (mm)					
	5 mm (Mean ± SD)			8 mm (Mean ± SD)		
	Hyper	Normal	Hypo	Hyper	Normal	Hypo
6Dr	1.43 ± 0.72	2.20±1.06	2.23±0.96	2.01 ± 1.03	3.49±1.64	3.25±1.25
7Mr	1.98 ± 1.17	1.91±0.41	1.41±0.65	3.51 ± 1.67	4.78±2.16	4.83±1.80
6Dl	1.64 ± 0.99	2.49±1.29	2.33±0.94	2.37 ± 1.28	3.74±1.95	3.93±1.54
7Ml	2.42± 1.53	3.92±2.29	3.41±1.76	4.12 ± 1.82	5.50±2.45	5.59±1.76

Abbreviations. Hyper, hyperdivergent individuals; Normal, normodivergent individuals; Hypo, hypodivergent individuals; 6Dr, distal root of the first molar right; 7Mr, mesial root of the second molar right; 6Dl, distal root of the first molar left; 7Ml, mesial root of the second molar left; SD: standard deviation.
